# Supplementary material for: Development of novel optical character recognition system to reduce recording time for vital signs and prescriptions: A simulation-based study
Source: PLoS One. 2024 Jan 19;19(1):e0296319. doi: 10.1371/journal.pone.0296319 (PMC10798482; doi:10.1371/journal.pone.0296319)
Supplement: S1 Fig — Panel A. Vital signs within the normal state. Panel B. Vital signs in an abnormal state. Panel C. Vital signs in the shock state. (PDF) [file pone.0296319.s003.pdf]

S1 Fig. Sample pictures of vital sign monitoring.

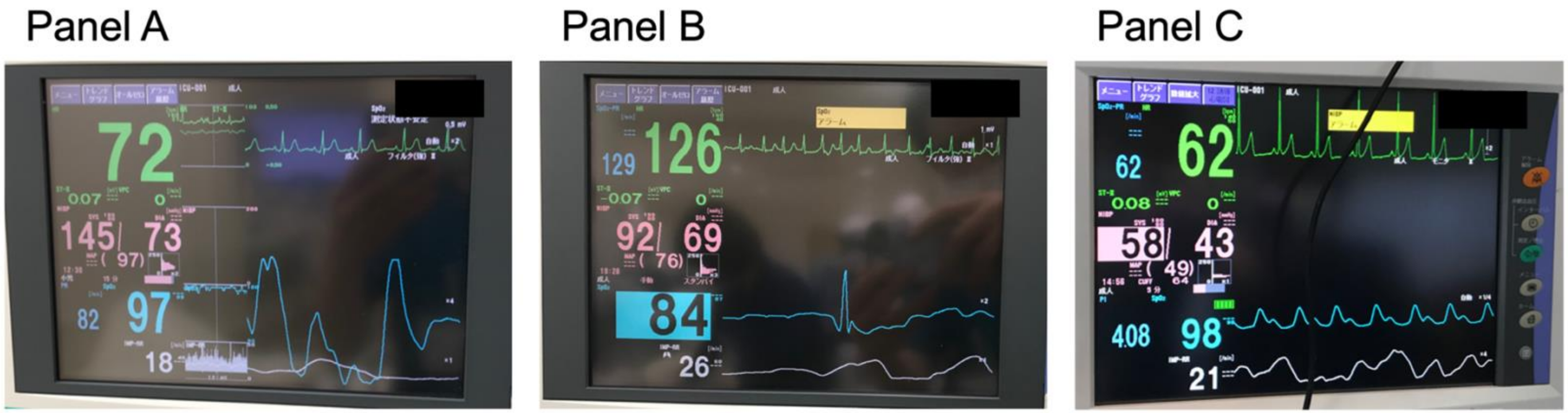

Panel A. Vital signs within the normal state: Heart rate, 72 beats/min; blood pressure, 145/73 mmHg SpO2, 97%; respiratory rate, 18/min. (Total number of characters to count for error rate calculation: 11).

Panel B. Vital signs in an abnormal state: heart rate, 126 beats/min; blood pressure, 92/69 mmHg; SpO2, 84%; respiratory rate, 26/min. (Total number of characters to count for error rate calculation: 11).

Panel C. Vital signs in the shock state: heart rate, 62 beats/min; blood pressure, 58/42 mmHg; SpO2, 98%; respiratory rate, 21/min. (Total number of characters to count for error rate calculation: 10)
