# Supplementary material for: Development of novel optical character recognition system to reduce recording time for vital signs and prescriptions: A simulation-based study
Source: PLoS One. 2024 Jan 19;19(1):e0296319. doi: 10.1371/journal.pone.0296319 (PMC10798482; doi:10.1371/journal.pone.0296319)
Supplement: S2 Fig — Prescription A (two medications; total number of characters to count for error rate calculation: 32). Prescription B (four medications; total number of characters to count for error rate calculation: 55). Prescription C (six medications; total number of characters to count for error rate calculation: 76). (PDF) [file pone.0296319.s004.pdf]

S2 Fig. Sample pictures of prescriptions in pharmacy notebooks

Prescription A (two medications; total number of characters to count for error rate calculation: 32**)**

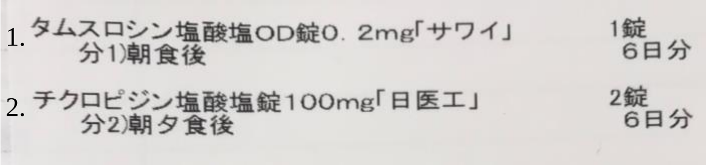

1. タムスロシン塩酸塩OD錠0. 2mg「サワイ」 1錠
分1)朝食後 6日分
2. チクロピジン塩酸塩錠100mg「日医工」 2錠
分2)朝夕食後 6日分

1. Tamsulosin Hydrochloride OD tablet 0.2mg 1tablet
   Once a day, after breakfast for six days
2. Ticlopidine Hydrochloride tablet 100mg 1tablet
   Twice a day, after breakfast, and dinner for 6 days

Prescription B (four medications; total number of characters to count for error rate calculation: 55**)**

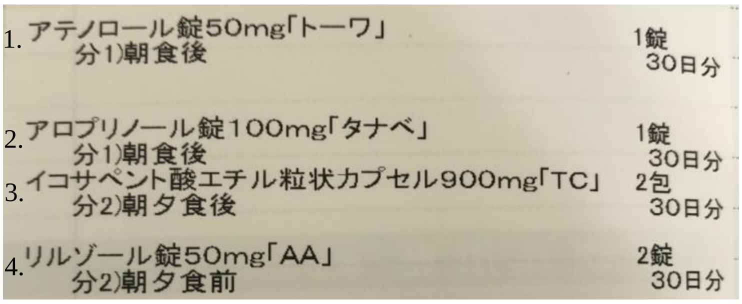

1. アテノロール錠50mg「トーワ」 1錠
分1)朝食後 30日分
2. アロプリノール錠100mg「タナベ」 1錠
分1)朝食後 30日分
3. イコサペント酸エチル粒状カプセル900mg「TC」 2包
分2)朝夕食後 30日分
4. リルゾール錠50mg「AA」 2錠
分2)朝夕食前 30日分

1. Atenolol tablet 50mg 1 tablet
   Once a day, breakfast was given for 30 days.
2. Allopurinol tablet 100mg 1 tablet
   Once a day, breakfast was given for 30 days.
3. Ethyl icosapentate granular capsules 900 mg 2 capsules
   Twice a day, after breakfast and dinner for 30 days
4. Riluzole tablet 50mg 2 tablets
   Twice a day, after breakfast and dinner for 30 days

Prescription C (six medications; total number of characters to count for error rate calculation: 76**)**

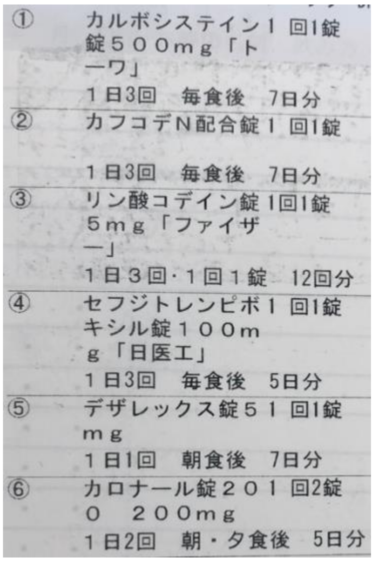

① カルボシステイン錠５００mg「トーワ」 1回1錠
1日3回　毎食後　7日分

② カフコデN配合錠 1回1錠
1日3回　毎食後　7日分

③ リン酸コデイン錠５mg「ファイザー」 1回1錠
1日３回・１回１錠　12回分

④ セフジトレンピボキシル錠１００mg「日医工」 1回1錠
1日3回　毎食後　5日分

⑤ デザレックス錠５mg 1回1錠
1日1回　朝食後　7日分

⑥ カロナール錠２００　２００mg 1回2錠
1日2回　朝・夕食後　5日分

1. Carbocysteine tablets 500 mg 1 tablet at a time
   Three times a day, after meals for 7 days
2. Coughcode-N Combination Tablets 1 tablet at a time.
   Three times a day, after meals for 7 days
3. Codeine phosphate 5 mg 1 tablet at a time
   Three times a day, 10 times
4. Cefditoren pivoxil tablet 100mg 1 tablet at a time
   Three times a day, after meals for 5 days
5. Desalex 5 mg 1 tablet at a time
   Once a day, after breakfast for seven days
6. Two 200 mg 2 tablet at a time.
   Twice a day, after breakfast, and dinner for 5 days
