# Supplementary material for: Development of novel optical character recognition system to reduce recording time for vital signs and prescriptions: A simulation-based study
Source: PLoS One. 2024 Jan 19;19(1):e0296319. doi: 10.1371/journal.pone.0296319 (PMC10798482; doi:10.1371/journal.pone.0296319)
Supplement: S3 Fig — (PDF) [file pone.0296319.s005.pdf]

S3 Fig. Recording time by profession

(A) Vital signs and (B) prescription

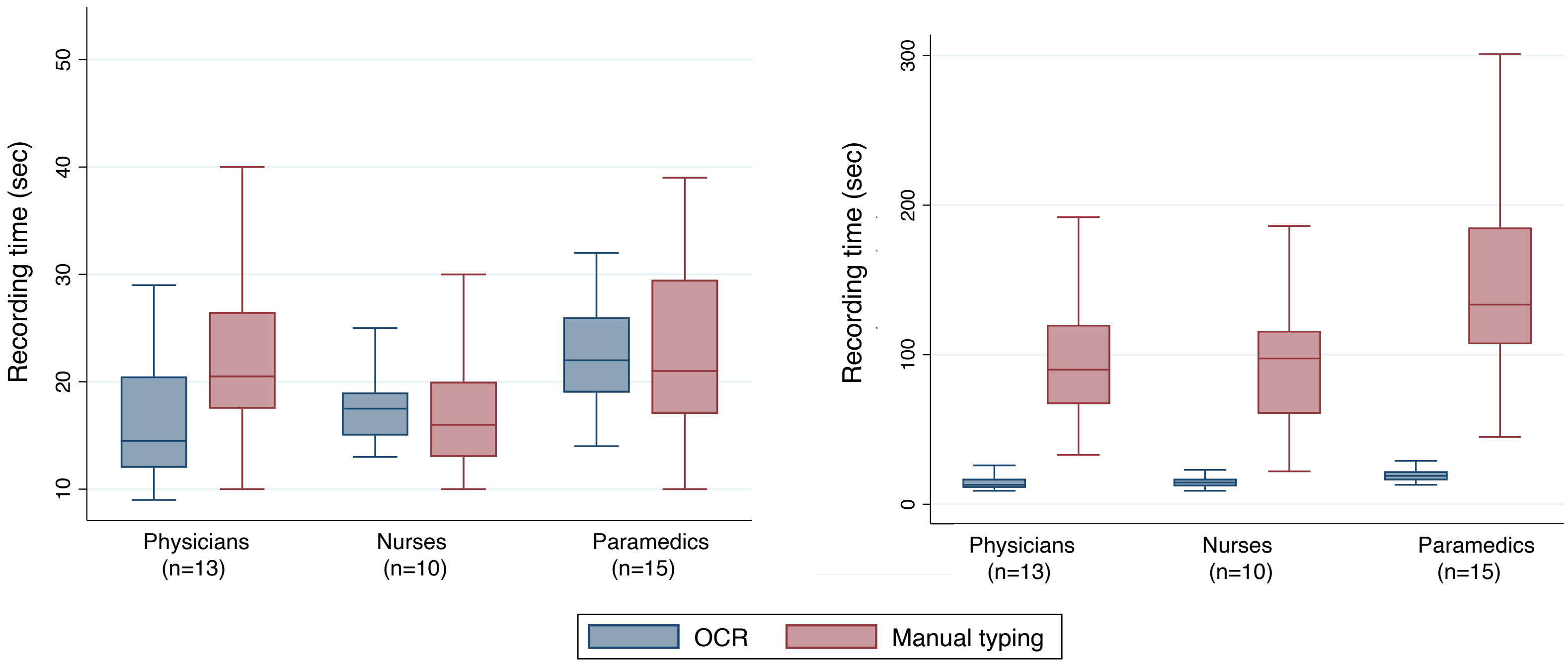

Abbreviations: OCR, optical character recognition
