# Supplementary material for: Development of novel optical character recognition system to reduce recording time for vital signs and prescriptions: A simulation-based study
Source: PLoS One. 2024 Jan 19;19(1):e0296319. doi: 10.1371/journal.pone.0296319 (PMC10798482; doi:10.1371/journal.pone.0296319)
Supplement: S4 Fig — (PDF) [file pone.0296319.s006.pdf]

S4 Fig. Recording time by Internet transmission speed (megabits per second)

(A) Vital signs

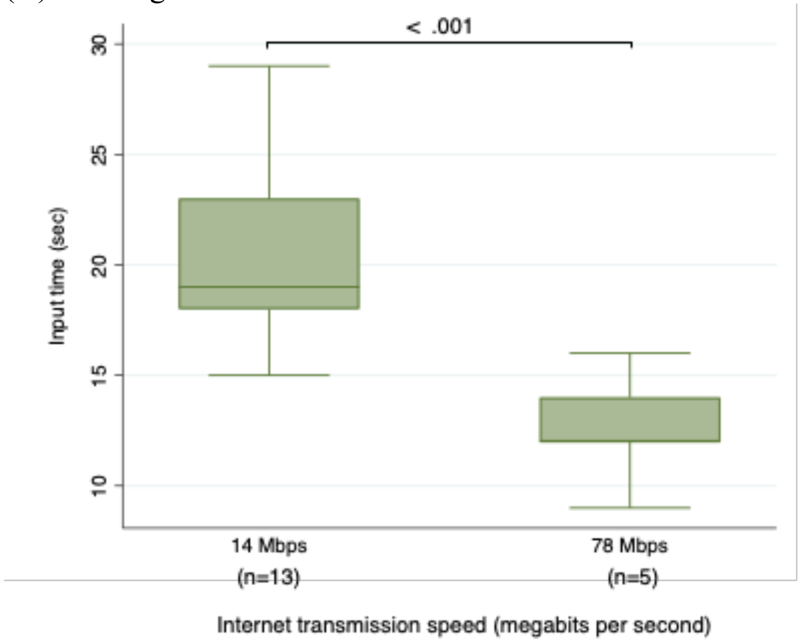

and (B) prescription

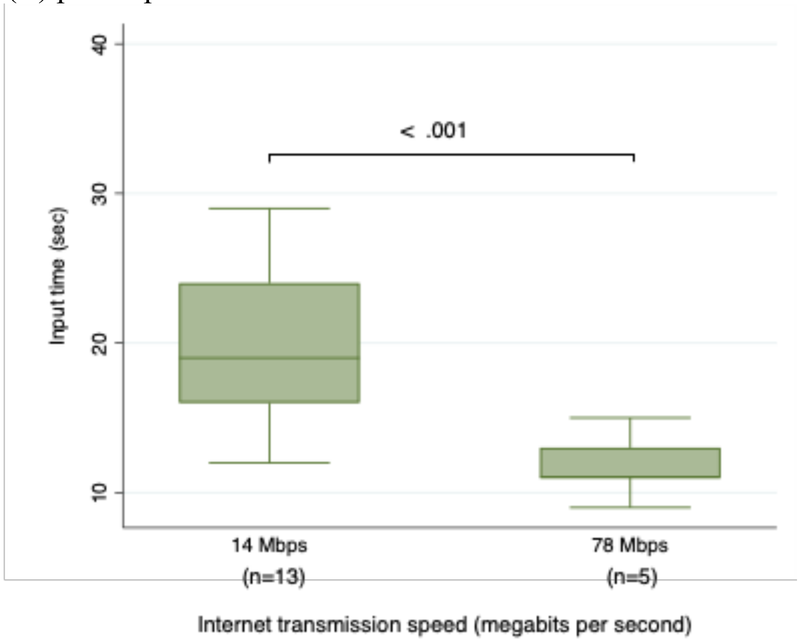

Abbreviations: Mbps, megabits per second.
