# Supplementary material for: Development of novel optical character recognition system to reduce recording time for vital signs and prescriptions: A simulation-based study
Source: PLoS One. 2024 Jan 19;19(1):e0296319. doi: 10.1371/journal.pone.0296319 (PMC10798482; doi:10.1371/journal.pone.0296319)
Supplement: S1 Table — (PDF) [file pone.0296319.s007.pdf]

S1 Table 1. Differences in recording time between the optical character recognition and manual typing groups (n=38)

Recording target	OCR group (n=38)	Manual typing group (n=38)	P value [a]
Vital signs on the monitor			
Overall	19 (15–23)	19 (15–26)	.15
Severity of the case: Normal state	21 (17–26)	23 (18–31)	.11
Abnormal state	18 (15–23)	18 (16–24)	.52
Shock state	18 (14–22)	18 (13–23)	.92
Prescription lists			
Overall	16 (13–20)	108 (72–143)	< .001
Number of medications on the list: 2	15 (13–19)	79 (59–112)	< .001
4	15 (12–18)	95 (70–126)	< .001
6	18 (14–21)	144 (112–187)	< .001

Footnote: Data are presented as median with interquartile range (IQR).

We excluded the following paired data from the analysis: five pairs from overall vital signs cases, two pairs from normal cases, one pair from abnormal cases, two pairs from shock state cases, nine pairs from overall prescription cases, six pairs from four medication cases, and three pairs from six medication cases.

[a] Wilcoxon signed-rank test.

Abbreviations: OCR, optical character recognition; IQR, interquartile range
