# Supplementary material for: Development of novel optical character recognition system to reduce recording time for vital signs and prescriptions: A simulation-based study
Source: PLoS One. 2024 Jan 19;19(1):e0296319. doi: 10.1371/journal.pone.0296319 (PMC10798482; doi:10.1371/journal.pone.0296319)
Supplement: S2 Table — (PDF) [file pone.0296319.s008.pdf]

S2 Table. Differences in recording time by Internet transmission speed (megabits per second)

	Internet Transmission speed		
Recording target	14 Mbps (n = 13) [a]	78 Mbps (n=5) [b]	P value[c]
Vital signs on the monitor	19 (18-23)	12 (12-14)	< .001
Prescription lists	19 (16-24)	11 (11-13)	< .001

Footnote: Data are presented as median with interquartile range (IQR).

[a]Data were available from 4 nurses and 9 paramedics.

[b]Data are available from five doctors

[c]Mann-Whitney U test

Abbreviations: IQR, interquartile range; Mbps, megabits per second
