# Supplementary material for: Development of novel optical character recognition system to reduce recording time for vital signs and prescriptions: A simulation-based study
Source: PLoS One. 2024 Jan 19;19(1):e0296319. doi: 10.1371/journal.pone.0296319 (PMC10798482; doi:10.1371/journal.pone.0296319)
Supplement: S3 Table — (PDF) [file pone.0296319.s009.pdf]

S3 Table. Differences in recording time between optical character recognition and manual typing and complete case analysis (n=23)

Recording target		OCR group (n=23)	Manual typing group (n=23)	P value [a]
Vital signs on the monitor				
Overall		18 (15-22)	18 (15-25)	.261
Severity of the case: Normal state		21 (17-26)	23 (18-31)	.206
Abnormal state		18 (15-23)	18 (16-24)	.563
Shock state		18 (14-22)	18 (13-23)	.988
Prescription lists				
Overall		16 (13-19)	111 (81-145)	< .001
Number of medications on the list:	2	16 (13-23)	83 (61-113)	< .001
	4	15 (13-19)	108 (78-133)	< .001
	6	17 (14-20)	144 (112-186)	< .001

Footnote: Data are presented as median with interquartile range (IQR).

[a] Wilcoxon signed-rank test.

Abbreviations: OCR, optical character recognition; IQR, interquartile range
