# Supplementary material for: Development of novel optical character recognition system to reduce recording time for vital signs and prescriptions: A simulation-based study
Source: PLoS One. 2024 Jan 19;19(1):e0296319. doi: 10.1371/journal.pone.0296319 (PMC10798482; doi:10.1371/journal.pone.0296319)
Supplement: Attachment — Submitted filename: PLOS ONE Review.docx [file pone.0296319.s010.docx]

MANUSCRIPT ID-

PONE-D-22-12113

TITLE:

Development of novel optical character recognition system to reduce recording time for

vital signs and prescriptions: A simulation-based study

Authors are suggested to read the mentioned points and incorporate the changes.

1. The abstract mentioned gives the clear idea about the work. But it should be written in a single paragraph and these headings should be explained later in the manuscript.
2. The abstract in the manuscript should be crisp.
3. Background study of the work should be clearly mentioned in the manuscript. It is expected the authors must add this to the paper.
4. Flow of paper is not well defined.
5. Figure 1 is about recording Flow, it is required to explain the steps in detail in content, it would be better to have a clear understanding of the workflow too.
6. Figure 1 Panel B seems of low quality. Make the figures in higher resolution and the labels can be readable.
7. Give the reference of the data used in Table 1 & 2 if it is taken from some other work or govt. data.
8. Kindly add the authentic reference of the codes of existing models taken in the manuscript.
9. Overall, the manuscript requires major revisions. There are few things to be verified by at author’s end. Like proper citation and referencing wherever the data and facts are mentioned.
10. Similarly, authors are advised to check the grammar as well as spellings. Like in line number 229 on page 12 *patent* is mentioned instead of *patient.*
11. Authors should go through journal template also.

Editor’s Comment:

1. This paper requires major revisions.
2. Editor kindly check the scope of journal with paper.
