# Supplementary material for: Development of novel optical character recognition system to reduce recording time for vital signs and prescriptions: A simulation-based study
Source: PLoS One. 2024 Jan 19;19(1):e0296319. doi: 10.1371/journal.pone.0296319 (PMC10798482; doi:10.1371/journal.pone.0296319)
Supplement: Attachment — Submitted filename: Response_to_Reviewers.docx [file pone.0296319.s011.docx]

Response to Reviewers

Title: Development of novel optical character recognition system to reduce recording time for

vital signs and prescriptions: a simulation-based study

Manuscript ID: PONE-D-22-12113

Revision Version: 1

Editor’s Decision Received Date: Jul 28, 2023

Revision Submission Date: Sep 10, 2023

Author Response 1^st^ revision

Reviewer 1

Reviewer Comments:

**1. The abstract mentioned gives the clear idea about the work. But it should be written in a single paragraph and these headings should be explained later in the manuscript. The abstract in the manuscript should be crisp.**

Author Response: We have removed the headings and condensed the abstract into a single paragraph to make it more concise and reflective of the study's key findings.

**2. Background study of the work should be clearly mentioned in the manuscript. It is expected the authors must add this to the paper.**

Author Response: Several studies have evaluated optical character recognition (OCR) capabilities; however, most focused on paper-based evaluations [2,3]. To our knowledge, only one study has explored OCR for monitor images. Furthermore, the previous study did not consider its use in the pre-hospital setting [3]. As such, our study focused on its use in the pre-hospital field by emergency medical services.

We have revised the **Introduction** section as follows:

P**age 3, para 1**: “Despite the evolution of clinical technologies, traditional methods of information sharing, including paper-based medical records, remain prevalent in many settings. This is especially evident in prehospital settings, emergency departments, and disaster sites. While the advancement and widespread adoption of mobile devices have enhanced healthcare, particularly in prehospital settings [1], the handover process from prehospital care to emergency departments often still depends on paper documents or manual input into smart device interfaces.”

.

P**age 3, para 3** “Previous studies explored the optical character recognition (OCR) technology used to capture vital signs from a paper-based encounter or commercial devices, such as oximeters and thermometers [2,3]. However, these studies did not account for the unique conditions of prehospital settings. Such environments present distinct challenges, including time constraints and the emergency medical personnel requirement to use the system while providing treatment.”

**3. Flow of paper is not well defined.**

Author Response: First, we revised the **Introduction** section, thus providing a clearer understanding of the study background and its objectives as outlined above. Second, **Figure 1** was restructured to offer a more intuitive depiction of the research flow. Finally, we revised the **Discussion** section to enhance the clarity of our research findings and describe potential future directions.

**4. Figure 1 is about recording Flow, it is required to explain the steps in detail in content, it would be better to have a clear understanding of the workflow too.**

Author Response: We have provided a detailed explanation of the steps in the recording flow presented in **Figure 1**.

**5. Figure 1 Panel B seems of low quality. Make the figures in higher resolution and the labels can be readable.**

Author Response: We have improved the resolution and readability of **Figure 1 Panel B** accordingly.

**6. Give the reference of the data used in Table 1 & 2 if it is taken from some other work or govt. data.**

Author Response: The data were specifically obtained only for this study on-site at each hospital. We did not use data from other studies or publicly available data.

**7. Kindly add the authentic reference of the codes of existing models taken in the manuscript.**

Author Response: We cannot disclose the code for the OCR engine. For more details about the development of OCR itself including the codes, please directly contact TXP Medical Co., Ltd. (https://txpmedical.jp/en/contact).

**8. Overall, the manuscript requires major revisions. There are few things to be verified by at author’s end. Like proper citation and referencing wherever the data and facts are mentioned.**

Author Response: We have revised the manuscript accordingly. The major revised points are as follows:

- As mentioned in the responses to your comments 1–3, we have revised the abstract, Introduction, and Discussion section.
- We have added two additional citations on OCR use in clinical settings to the Introduction section [2,3].
- We have now mentioned recent studies that show optical character recognition/natural language processing (NLP) hybrid usage significantly improving operational efficiency of the data extraction to the Discussion section [12-13] (page 15, para 1).

**9. Similarly, authors are advised to check the grammar as well as spellings. Like in line number 229 on page 12 patent is mentioned instead of patient.**

Author Response: We have repeated the process of checking and editing the English by a native speaker multiple times to make corrections, including correcting "patient" on page 12, line 229 (now **Page 12, para 1**).

**10. Authors should go through journal template also.**

Author Response: We have thoroughly reviewed the journal's formatting guidelines and ensured that our manuscript complies with them.

Reviewer 2

Reviewer Comments:

**1. As mentioned in line no 72 "Using the iphone"; isnt the system applicable on any other smartphone other than iphone.**

Author Response: In this study, we used only iPhones and iPads due to cost and resource constraints. Although the OCR system is not yet available on the Android platform, we are in the process of planning its implementation. We included the fact that only iPhones were used in this experiment to the Limitation section (**Page 13, para 4.**)

**2.The number of participants taken for sensitivity analysis are very less. How any one can identify the appropriate results with less no of input values provided. Explain**

Author Response: Sensitivity or subgroup analyses were not primary analyses but were conducted primarily to identify trends or generate hypotheses. This sensitivity analysis obtained similar results as the primary analysis. For the next phase of OCR implementation, we should determine the sample size based on these results. Evaluations in various environments will be warranted as we mentioned in the Limitation section (**Page 14, para 1**).

**3.Abstract should be more clear for reader's perspective.**

Author Response: We have revised the abstract in the manuscript for clarity.

**4.Authors have provided a comparison with earlier works almost 5 years back, which cannot support this work as per the current scenario.**

Author Response: We have now cited an article from 2021 [3] and revised the Introduction section (**Page 3, para 3**). As we mentioned in the response to Reviewer 1's comment 2, the previous study did not consider the use in the prehospital setting. They examined the use of OCR for commercial devices such as thermometers or wearable devices at home. On the other hand, our study focused on its use in the prehospital field by emergency medical services to document vital signs and prescription lists.

**5.Authors should write the main contributions of the work properly. Authors have written architectural details instead of writing the main contributions.**

Author Response: KL designed the experiment, and SS carried out the experiment and analyzed the data. TS provided the devices and applications used in this experiment. SS and KL wrote the manuscript with support from SW and TS. TG supervised the project. All authors had full access to all study data and take responsibility for its integrity and analytical accuracy.

**6.Authors should take care of many typos and grammatical mistakes in the manuscript.**

Author Response: We have carefully proofread the manuscript and corrected all grammatical errors and misspellings.

**7.A careful proofreading is required to improve the readability of the paper.**

Author Response: We have comprehensively reviewed the manuscript to enhance its readability.

**8.Authors didn’t include any references from year 2023. An introduction is an important road map for the paper that should be consists of an opening hook to catch the researcher's attention, relevant background study, and a concrete statement that presents main argument but your introduction lacks these fundamentals, especially relevant background studies. This related work is just listed out without comparing the relationship between this paper's model and them; only the method flow is introduced at the end; and the principle of the method is not explained. To make soundness of your study must include these latest related works (years 2022-2023).**

Author Response: We have added recent studies on OCR use in clinical settings [12-13].

We have revised the **Discussion** section as follows:

**Page 12, para 3:** “Finally, this OCR technology could be applied to various other clinical settings, such as general screening at the scheduled hospital admission and a patient’s first visit to a clinic with no medical history. OCR/natural language processing (NLP) hybrid usage significantly improved data extraction efficiency [12,13]. The importance of OCR is likely to increase even further when used in conjunction with NLP in the future.”

”

**9.Mention the limitations and future works of the developed system elaborately.**

Author Response: As you kindly pointed out in your questions 1 and 2, we included the limited sample size and the fact that only iPhones were used in this experiment in the Limitations section (**Page 13, para 4**).

We now describe the limitations as follows: “Our study had several limitations. First, our data set included missing data and outliers within a small sample size, which could affect the interpretation of the results. However, excluding cases missing data did not affect the primary outcome of this study (**Table S3**). A larger sample size is required to validate the reliability of these results. Second, we used only six sample photographs in the experimental setting. The effect and error rate of the OCR must be verified in actual prehospital and clinical settings. Third, we used only iPhones in the experiment. Fourth, recruiting participants on a volunteer basis may have caused selection bias. Finally, although the pre-calculated sample size was achieved in this study, the number of participants and occupations involved remained limited. For instance, medical clerks, who often help to document medical information, did not participate in this study. As a next step, we will incorporate this technology in clinical practice, mainly in the prehospital or emergency care settings, and investigate its reliability and effectiveness in future studies.

”
