# Supplementary material for: Development of novel optical character recognition system to reduce recording time for vital signs and prescriptions: A simulation-based study
Source: PLoS One. 2024 Jan 19;19(1):e0296319. doi: 10.1371/journal.pone.0296319 (PMC10798482; doi:10.1371/journal.pone.0296319)
Supplement: Attachment — Submitted filename: review 2_Plosone.docx [file pone.0296319.s012.docx]

**Minor Revisions**

- The article must present a scientific study that is technically competent and has evidence to back up its findings.
- Rigidly designed experiments with the right controls, replication, and sample sizes must have been used..
- The conclusions must be drawn appropriately based on the data presented.
- Authors have provided a comparison with earlier works almost 5 years back, which cannot support this work as per the current scenario.
- A careful proofreading is required to improve the readability of the paper.
- Latest References not included in the paper.
