# Supplementary material for: Development of novel optical character recognition system to reduce recording time for vital signs and prescriptions: A simulation-based study
Source: PLoS One. 2024 Jan 19;19(1):e0296319. doi: 10.1371/journal.pone.0296319 (PMC10798482; doi:10.1371/journal.pone.0296319)
Supplement: Attachment — Submitted filename: 20231107_Response_to_Reviewers.docx [file pone.0296319.s013.docx]

Response to Reviewers

Title: Development of novel optical character recognition system to reduce recording time for

vital signs and prescriptions: A simulation-based study

Manuscript ID: PONE-D-22-12113

Revision Version: 2

Editor’s Decision Received Date: Jul 28, 2023

Revision Submission Date: Sep 9, 2023

Second Revision Submission Date: Nov 7, 2023

Author Response 2^nd^ revision

Reviewer #1

Reviewer Comments:

**1. The article must present a scientific study that is technically competent and has evidence to back up its findings.**

Author Response: Thank you for the comment. The research design of this study was carefully considered. We have addressed this in our response to comment 2. Following your comment, we have also included two references to substantiate our findings following your comment no. 4.

**2. Rigidly designed experiments with the right controls, replication, and sample sizes must have been used.**

Author Response: Thank you for this comment. In this study, we used intra-person comparisons because the recording time of clinical information inherently involves inter-person differences. Therefore, the control group in this study was appropriate for our research objectives. Regarding sample size, we conducted a post hoc analysis to ensure that the study was adequately powered. For the vital signs data, we considered a scenario in which manual input required a mean of 30 s (standard deviation [SD], 10 s). When comparing this to a mean of 25 s for OCR, with a significance level (α) set at 0.05 and a power (β) of 0.80, our calculations indicated that a sample size of 32 individuals would be necessary. For recording the prescriptions, we considered a scenario in which OCR reduces input time from a mean of 100 s (SD, 30) to 50 s, with an α set at 0.05 and a β of 0.80. This calculation indicated that a sample size of 12 individuals was sufficient. Overall, based on these post-host estimates, our sample size had sufficient statistical power to detect significant differences. Although we did not conduct repeated measurements for each participant, we acknowledge this aspect in the Limitations section of the manuscript. Accordingly, we revised the manuscript as follows:

**Methods section (Page 7, para 2)**: “We also conducted a post hoc analysis to ensure that the study was adequately powered. For vital signs, assuming an average input time of 30 s (SD, 10 s) for manual typing and 25 s for OCR, with a significance level (α) set at 0.05 and a power (β) of 0.80, the required sample size was 32 individuals. For prescriptions, we considered a scenario in which OCR reduces input time from an average of 100 s (SD, 30 s) to 50 s, with an α set at 0.05 and a β of 0.80, a sample size of 12 individuals was sufficient.”

**Methods section (Page 8, para 1):** “Intra-person comparisons were used because the recording time of clinical information inherently involves inter-person differences.”

**Limitations section (Page 15, para 2)**: “In addition, we did not conduct repeated measurements for each participant, which may have limited the replicability of our findings.”

**3. The conclusions must be drawn appropriately based on the data presented.**

Author Response: Thank you for the comment. As you suggested, we have removed the unclear sentence from the **Conclusion** section to focus on the data presented:

**Page 15, para 3**: “Using an OCR developed by machine learning with pattern recognition significantly reduced the time required to record prescription lists. ~~OCR accurately translates clinical information (vital signs and prescription lists) into digitized data as an alternative to manual typing.~~ Further clinical studies are warranted to confirm the effects of OCR systems on patient outcomes.”

**4. Authors have provided a comparison with earlier works almost 5 years back, which cannot support this work as per the current scenario.**

Author Response: We are sincerely grateful for your comment.

We conducted a PubMed search for "optical character recognition" and found 119 articles published since 2018. However, none were related to emergency medicine, our research field. If there are specific references that should be included in relation to this aspect, we would be happy to add them. A review of the abstracts of the 119 articles identified that the following studies relevant to our research:

- Liu W, Yu X, Wang J, Zhou T, Yu T, Chen X, et al. Improving kidney outcomes in patients with nondiabetic chronic kidney disease through an artificial intelligence-based health coaching mobile app: a retrospective cohort study. JMIR Mhealth Uhealth. 2023;11:e45531.

This study assessed the effect of a mobile app-based intelligent care system with OCR on kidney outcomes in patients with CKD. This earlier study analyzed data from the KidneyOnline system in China and found a significant reduction in kidney-related issues in patients using the intelligent care system. We have mentioned this article as reference no. 4 in the **Introduction** section.

**Page 3, para 3: “**A mobile app–based intelligent care system, including OCR, has the potential to reduce health-related issues in patients with chronic kidney disease [4].”

- Lee SY, Park JH, Yoon J, Lee JY. A validation study of a deep learning-based doping drug text recognition system to ensure safe drug use among athletes. Healthcare (Basel). 2023;11:1769.

This study developed an English doping drug recognition system using deep learning–based OCR and discussed solutions to reduce reading errors in OCR. We have added this study as reference no. 9 and the following sentences to the **Discussion** section:

**Page 13, para 4**: “Errors that occur with OCR use are often attributed to the photo-resolution [9]. Introducing a system to detect and request the re-uploading of low-resolution photos can help collect training data to mitigate these errors.”

**5. A careful proofreading is required to improve the readability of the paper.**

Author Response: Thank you for the comment. We carefully proofread the manuscript to ensure its readability and have attached an English proofreading certificate.

**6. Latest References not included in the paper.**

Author Response: As mentioned in the response to comment no. 4, we have added the abovementioned papers to the references. We appreciate your suggestion to prevent missing important background on this research topic.
